# Evaluating the Safe Steps for De-escalation: A protocol for a mixed concurrent control study in acute mental health units

**DOI:** 10.1371/journal.pone.0325558

**Published:** 2025-06-03

**Authors:** Esario IV Daguman, Alison Taylor, Matthew Flowers, Dane Owen, Allyson Wilson, Richard Lakeman, Marie Hutchinson

**Affiliations:** 1 Faculty of Health, Southern Cross University, Coffs Harbour, New South Wales, Australia; 2 Integrated Mental Health, Alcohol and Other Drugs, Coffs Harbour Base Hospital, Coffs Harbour, New South Wales, Australia; Dilla University College of Health Sciences, ETHIOPIA

## Abstract

There is a shared goal of organising reform efforts in mental health services to eliminate restrictive practices and improve therapeutic relationships. However, evidence on high-quality, culturally safe, co-produced, and strengths-based interventions and evaluations is limited, especially for complex interventions centred on therapeutic responding. In response, a multi-centre, mixed concurrent control study is underway to evaluate the Safe Steps for De-escalation, a multi-component intervention focused on a structured framework for mental health nurses’ therapeutic responses to emotional distress and interpersonal conflict in acute adult mental health inpatient units. The aims of this evaluation were: 1) What is the effectiveness of Safe Steps in reducing restrictive practice events and duration and physical injuries? 2) Does Safe Steps improve people’s service experience, perceived staff action towards violence prevention, and nurses’ professional quality of life and emotionally intelligent workplace behaviours? 3) What factors influence the successful implementation of Safe Steps? It is hypothesised that: a) intervention sites will demonstrate more significant decreases in restrictive practice events and duration and physical injuries, compared to within-group baseline and control group, and b) measures of people’s experiences and perceptions and nurses’ outcomes and behaviours will improve, compared to within-group baseline. Safe Steps has three components: i) a structured de-escalation framework, ii) an in-person and online training programme, and iii) a regular conduct of strengths-based, data-informed restrictive practice review meetings. The control group will be usual care. Other outcomes include nursing intervention clusters, their associations with various outcomes, and factors influencing intervention implementation and restrictive practice use. There is no randomisation, but inverse probability weighting will be applied. The sample sizes were determined through power analyses and supporting evidence on saturation in qualitative research. Various quantitative and qualitative data treatments and measures will be undertaken to minimise research biases.

## Introduction

Restrictive practices are measures that limit a person’s [1] rights or freedom of movement [2]. In acute mental health services, a shared goal exists among people receiving care, carers, professionals, policymakers, advocates, and organisations to eliminate these restrictive practices and promote therapeutic relationships [3]. This goal has been recently strengthened, with calls for rights-based mental health systems, person-centred care, stigma reduction, greater inclusion, and an end to coercion [4]. However, the evidence on interventions supporting this goal remains limited in quality and considerations of culturally safe, co-produced, strengths-based, and complexity-informed approaches [5]. Beyond a few well-known programmes, most initiatives in adult inpatient settings also exist only in grey literature and are not well-described [6], with study designs and protocols not openly available.

Seclusion, physical restraint, and forced medication are restrictive practices that exemplify legal coercion [7]. These regulated and documented interventions are employed when less stringent measures have been exhausted in response to a perceived risk of harm [8]. There are differences in the language, meaning, regulation, monitoring, and prevalence of these practices in adult mental health inpatient services worldwide, including Australia [9]. Attitudes toward restrictive practices also vary, with many healthcare professionals, especially nurses, seeing them as unavoidable, as a last resort [10], as something that could not be fully eradicated from practice [11], and as apt within staffing constraints and limited available options [12]. Restrcitive practices are considered traumatic and are associated with a range of harms, including but not limited to isolation for Indigenous people [13] and disruption of therapeutic relationships between individuals receiving care and nurses [14].

Interventions and programmes have been developed and evaluated to offer alternatives to restrictive practices and elevate the value of therapeutic relationships. Examples include hospital unit redesign [15], staff education and training [16], sensory rooms [17], risk assessment [18,19], and multi-component interventions, such as the Safewards [20] and programmes [21,22] based on the Six Core Strategies to Reduce Seclusion and Restraint [23,24]. However, many of these interventions have a mixed impact, have been inconsistently reported, have not been evaluated against aggression and physical injury outcomes, have been assessed against composite measures (rather than separate seclusion, restraint, and sedation medication outcomes), and have typically been analysed with no account for confounds [25]. No advanced statistical techniques, such as matching or weighting, have been used to adjust for differences between comparator groups in place of randomisation, which is often difficult to achieve in this field [26]. More importantly, individual components of complex solutions like Safewards have never been tested in isolation [27]. There have been no attempts to understand what component drove the overall impact.

A similar state is seen in de-escalation interventions, despite being the first-choice response to emotional distress and interpersonal conflict [28]. In de-escalation training interventions, there are limitations to the: coverage of theories and components behind therapeutic responding, adaptations to individuals and cultures, quality of evaluation methodologies, evidence of effectiveness, and evaluation against sedation medication outcomes [29]. The Safewards de-escalation component, ‘Talk Down,’ highlights this gap; it has been criticised in the Australian context for being patronising and condescending, with some accounts describing its language as infantilising [30]. Moreover, it is unclear in many de-escalation interventions which specific components contribute to a framework’s success.

### The Safe Steps for De-escalation

In a regional adult mental health inpatient unit in New South Wales (NSW), Australia, a lack of structure to therapeutic responding was evident in the absence of a consistent content and approach to de-escalation training, the varying skill levels of nurses in managing distress and conflict, and the persistently high rates of physical restraint and seclusion [31]. In response, a new model was introduced in the unit in 2019 to monitor restrictive practices and up-skill nurses, and implement the 2017 NSW review recommendations on seclusion, restraint, and observation [32]. The model was implemented alongside strength-based review meetings, where nurses received feedback on how well they practice relational competencies. The reviews conducted between January 2019 and March 2020 were linked to a reduction in the use of seclusion (incidence rate ratios [IRR] = 0.37, 95% CI [0.24, 0.57], p < .001), compared to an equivalent timeframe before their implementation [33]. Measures of experienced support by the people receiving care in the unit also increased by 14% in 2019–2020, exceeding the 80% national benchmark [34].

This model is called the *Safe Steps for De-escalation*—or simply the *Safe Steps.* It is a clinician initiative centred on a four-step, user-friendly framework for therapeutic responding that begins with helping the person receiving care to identify the issue or problem, through to establishing expected behaviours. There is evidence for the structure and progression of therapeutic responses in the Safe Steps [35] that offers nurses a platform to exercise their authentic voice and intuitive competencies in supporting work towards making acute units safe. The Safe Steps targets nurses’ relational competencies to increase focus on the self-management of people receiving care and therapeutic relationships. Its implementation is supported by value-based training and regular data-informed restrictive practice review meetings.

The Safe Steps model has generated interest in mental health services across NSW, with training on the model recognised and recommended for implementation in other services. It has been further developed into a full programme that includes meaningful cultural consultation, peer and advocate involvement, and multi-professional input, as part of a multi-site translational research. Informing its further development is a range of knowledge bases on the value of therapeutic relationships, recovery, lived experience involvement, choice [36,37], trauma-informed care [38], mental health nursing emotional capabilities [38,39], mental health nurses’ professional identities [40,41], mental health nursing specialist education and training [42–44], and restorative approaches [45,46].

This protocol outlines a proposed series of studies evaluating the Safe Steps for De-escalation model, which was developed to address several gaps in the academic mental health literature. The model offers a structured approach to therapeutic responding, grounded in content, input, and participation from diverse key stakeholders, from intervention design, implementation, through to evaluation. The proposed evaluation addresses three core objectives and tests two overarching hypotheses. The first objective is to examine the effectiveness of the Safe Steps in reducing restrictive practice events, duration, and physical injuries. It was hypothesised that intervention units would show more significant reductions in IRR of restrictive practice events (defined as seclusion, physical restraint, and sedation via intramuscular injection [IMI]), estimates of restrictive practice duration (i.e., seclusion and physical restraint duration), and IRR of physical injuries, compared to control groups and within-group baseline. The second objective is to assess whether the Safe Steps improves people’s experience of services and perceived staff action on violence prevention, and nurses’ professional quality of life and demonstrated emotionally intelligent workplace behaviours. It was hypothesised that scores from these four measures at the final time point would be significantly higher than at baseline. Lastly, the third objective is to explore factors influencing the successful, pragmatic implementation of the Safe Steps.

## Materials and methods

### Design

**Fig 1:**
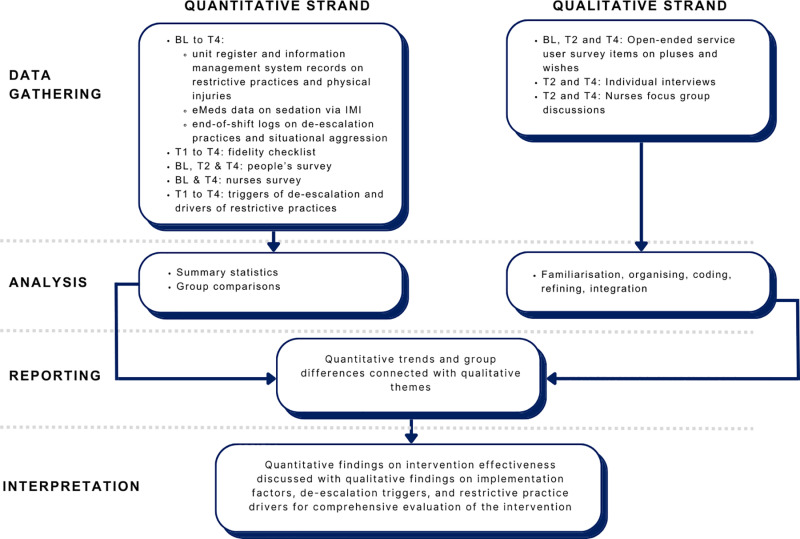
Concurrent mixed methods design diagram.

The current study has a mixed concurrent control design, where qualitative and quantitative data will be gathered simultaneously, analysed separately, and then integrated during the reporting and interpretation stages of the study [47]. Quantitative data will be collected to test the hypotheses that help predict the impacts of the intervention (see Fig 1). In contrast, qualitative data will help examine the underlying factors influencing the pragmatic implementation and explain the impacts of the intervention. This mixed-methods design was selected for its rigour and comprehensive approach to understanding a complex intervention, which enables integration across data sources to enhance the validity and credibility of the evaluation [48]. Including a control component addresses the standard limitation of intervention studies on reducing restrictive practices, which often lack control groups [21,26].

**Fig 2:**
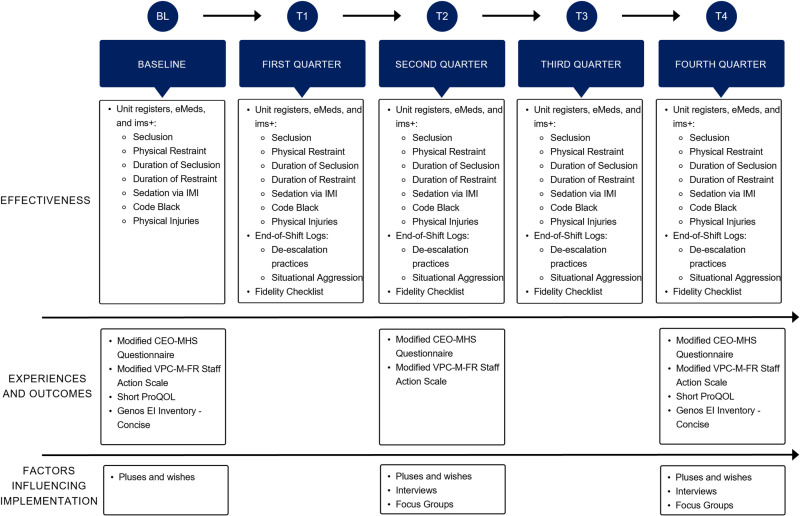
Outcomes and data gathering timeline. CEO-MHS = Consumer Evaluation of Mental Health Services; EI = emotional intelligence; eMeds = electronic medication management system; IMI = intramuscular injection; ims+ = incident management system; ProQOL = Professional Quality of Life; VPC-M-FR = Modified Violence Prevention Climate.

The effectiveness of the Safe Steps will be evaluated in three adult mental health inpatient units, as using multiple sites and varying the setting and context reduces the risk that findings are limited to the experimental setting [49]. Between March 2023 and April 2025, baseline and one-year follow-up outcomes will be assessed (see Fig 2), as implementation efforts on reducing restrictive practices are said to be consolidated within 12 months [50]. Four quarterly time points will be applied to align with the phased deployment of the Safe Steps’ training component, which would allow for the assessment of short-, medium-, and long-term outcomes and prevent misjudging the intervention’s effectiveness based on insufficient timeframes [51].

There will be three forms of comparisons within the current evaluation. Between- i) and within-group ii) comparisons will be applied to test the first and second sets of hypotheses. The third will be a between-cluster comparison iii), intended to identify the most active co-occurring therapeutic and nursing responses (clusters) contributing to the Safe Steps’ overall impact. The first set of hypotheses is that implementation units will exhibit greater reductions in the IRR for restrictive practice events, the estimate of restrictive practice duration, and the IRR for physical injuries, compared to control groups and their baseline levels. The second set of hypotheses is that measures of service experience, perceived staff action towards violence prevention, nurses’ professional quality of life, and nurses’ perceived demonstration of emotionally intelligent workplace behaviours at the final time point would be significantly higher than at baseline. The between-group comparisons will be conducted exclusively to address the first objective, as these analyses will depend on accessible routine reports on restrictive practices and physical injuries selected for this purpose. Australian public acute mental health services are legally mandated to report on seclusion and physical restraint events and duration as key performance indicators [52]. Events of physical injuries and sedation via IMI are also routinely documented in established information management systems. These routine measures will help minimise performance and detection bias associated with the lack of blinding. Conversely, conducting a between-group comparison on non-routine measures could be cost-prohibitive and challenging to implement, as exemplified by unmet sample size requirements in previous studies [27]. This evaluation will have no randomisation, although covariate balancing will be applied to between- and within-group comparisons in testing the first set of hypotheses.

The current study was informed by Skivington and colleagues’ complexity framework [53]. This framework proposes that complex intervention research can use a multiple-model view, where there can be a study on intervention effectiveness, an exploration of concepts, or an adoption of a systems approach, depending on what adds the most value. The framework balances precision in research with real-world complexity, focusing on how interventions work, their broader impact, and how findings can support everyday practice. This framework proposes that evaluation and implementation can happen simultaneously or iteratively, with interventions influenced by their implementation context [54], which has been instrumental in accounting for changes in mental health practice [55]. The non-linear nature of complex intervention development, evaluation, and implementation is reflected in the process of communicating this protocol. This protocol was developed to be published before study commencement, although iterative learning during implementation, alongside the staged nature of ethical approvals, led to amendments, particularly in the data analysis plan, in the middle of the study implementation and evaluation. As a result, the protocol was submitted for publication consideration after pragmatic implementation began. All procedures adhere to the approved protocol, and this version reflects updates following ethics approval of those changes.

The current study corresponds to the evaluation and implementation phases of the complexity framework [53], which supports the determination of an intervention’s effectiveness and what facilitates or blocks the attainment of impacts, respectively. This study’s first and second research objectives relate to the framework’s evaluation phase. In contrast, the third question corresponds to the implementation phase. Context and stakeholder engagement are core elements of the framework guiding this evaluation and implementation. Since the Safe Steps’ effectiveness may vary by site, only accessible confounders and covariates will be balanced in the analysis. At the same time, uncontrolled factors will be explained qualitatively. The evaluation will also gather perspectives from two main stakeholder groups on what supports implementation: nurses as the intervention’s target and individuals as care recipients.

**Fig 3:**
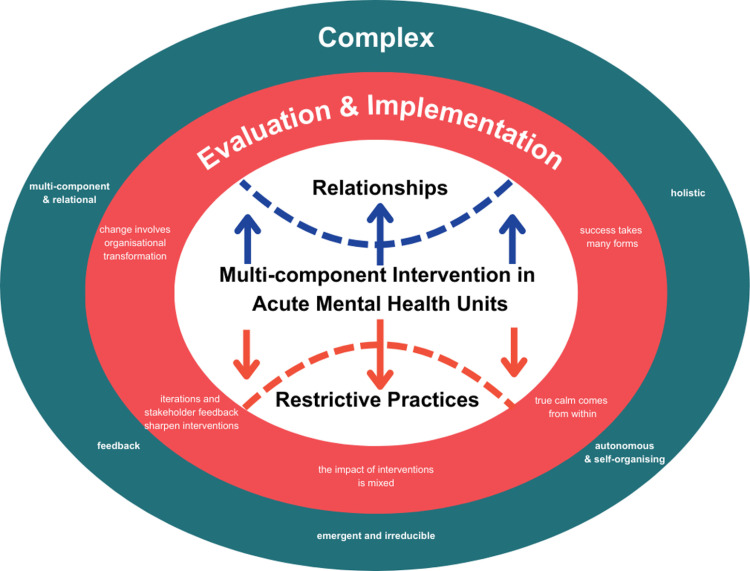
The guiding map.

The Safe Steps evaluation and implementation are guided by the complex adaptive system properties, including those described in Skivington and colleagues’ complexity framework [53], and pragmatic principles in research [56]. These paradigms give primacy to context, flexibility, and provisional understanding of a phenomenon [57]. However, neither is sufficient in isolation. Knowledge can often be verified step-by-step, but this is not always straightforward [58]. Instead, guiding statements can serve as a map for change. An overview of reviews on restrictive practice reduction efforts supports the need for pragmatic and complexity-informed approaches [5], where successful reforms were found to hinge on social, psychological, and research contexts. Despite these insights, it was also found in the overview that mental health research still tends to prioritise numerical outcomes, which can overlook the nuanced conditions that shape change. In response, the first author developed aphorism-like statements based on these two paradigms and the overview of review outcomes to inform the Safe Steps evaluation and implementation design. This framework is summarised in a single principle: Evaluation is as complex as deploying service changes. Fig 3 visualises this framework. Table 1 outlines the key points, what they mean, what complex adaptive system properties they represent, and what areas of the Safe Steps evaluation and implementation they inform.

**Table 1:** Summary of five key points guiding the Safe Steps evaluation and implementation.

Key point	What does this mean?	What property does it represent?	What does it inform?
Success takes many forms.	Positive outcomes in reducing restrictive practices and improving relationships can look different. What works for one service might not work for another.	Holism	Inclusion of outcomes beyond clinical frames, such as experiences of services, violence prevention climate, professional quality of life, and demonstrations of emotionally intelligent workplace behaviours
True calm comes from within.	There is no single solution to improving relationships and reducing restrictive practices. The heart of the complex intervention under study offers a structured approach. However, people receiving care and nurses may find other ways to achieve what works best for each individual.	Autonomy and Self-organisation	Capacity to consent in research assessment, selection of patient-rated experience measure with ‘empowerment’ subscale, qualitative data validation, opt-in-based intervention implementation, fidelity checks (i.e., the measure assesses fidelity to intervention components, rather than each step of the first component)
The impact of interventions is mixed.	Reducing restrictive practices might not always lead to a complete return to a previous state, as if reversing time. However, the process of change itself can offer unforeseen positive outcomes.	Emergence and Irreducibility	Implementation and evaluation timeframe, inclusion of other outcomes beyond usual seclusion and restraint (i.e., sedation via intramuscular injection, Code Blacks, and physical injuries), monitoring and feedback on nursing practice during reviews, qualitative strand intended to explore what supports implementation (and examine change process and any emergent impact of the intervention)
Iterations and stakeholder feedback sharpen interventions.	The intervention becomes more effective over time through a continuous adaptive process. People receiving care and nurses are central to this process. Their experiences and suggestions ensure the intervention is shaped to best meet their needs.	Feedback	The qualitative strand intended to explore what supports implementation (and examine participants’ feedback to improve the intervention), peer-led surveys, chosen patient-rated experience measure was developed with lived experience involvement, team-based approach in qualitative data analysis.
Change involves organisational transformation.	Arguing for change in a service is arguing for a change in many aspects of that service. Building strong relationships between people receiving care and nurses is vital in supporting these changes.	Multi-component and Relational	Work on emerging drivers of restrictive practices, the inclusion of situational aggression outcome, the inclusion of site champions during implementation, qualitative questions focused on nurse-individual relationships, qualitative strand intended to explore what supports implementation, three forms of comparisons (i.e., between- and within-group and between-cluster comparisons).

### Intervention

The Safe Steps for De-escalation is a one-year intervention that involves three core components and targets nurses’ relational competencies to increase focus on the self-management of people receiving care and therapeutic relationships. Within a year, nurses will receive phased training on the intervention’s first component and ongoing support and restrictive practice review meetings to integrate the learned framework of therapeutic responding into practice. The effectiveness of the intervention is assessed over this period, focusing on changes in a range of outcomes.

The Safe Steps’ first component refers to the i) four-step structured therapeutic response to emotional distress and interpersonal conflict. The structured framework provides a user-friendly, replicable approach that empowers nurses to implement de-escalation techniques, especially when under the additional load of high-stress environments. Each step is essential for supporting individuals receiving care in expressing their needs, feelings, and preferences, as well as identifying steps for self-management. This first component has been conceptualised by a clinician (MF) and refined through collaboration with a fellow clinician (AT).

Another component of the Safe Steps is an in-person and online training programme ii) on deploying the intervention’s first component and the value of emotionally intelligent, culturally safe, trauma-informed, and recovery-oriented engagement. The in-person training takes a phased approach to building nurses’ therapeutic and collaborative skills in aid of de-escalation. The online learning resource comprises four modules, complements the in-unit training, and underlines that restrictive practices should be considered a last resort. The training component was developed with bilateral and prolonged consultation with lived experience advocates, peer workers, Aboriginal health leads, mental health nurses and practitioners, researchers, and academics.

Regular conduct of data-driven, unit-level reflective practice iii) completes the Safe Steps intervention. It aims to sustain the intended service changes by allowing nurses to consider their relationships with individuals receiving care, their well-being, and technical competencies. The reflective practice incorporates a strengths-based review of aggregated data from completed de-escalation logs, including the successful therapeutic responses undertaken by the nurses. The design of the review component included input from peer workers and individuals with lived experience of mental distress and restrictive practices.

### Setting

The evaluation will be conducted across two NSW local health districts (LHDs). LHDs in NSW are health administrative regions with distinct budgets and governance under Australia’s universal healthcare framework, making up geographically based districts and three specialised networks. The intervention sites will consist of three declared adult mental health inpatient units, with a combined capacity of 75 beds, including ten designated for high observation. Operating 24 hours a day and situated within public hospitals, these units cater to varying levels of mental health treatment and observational needs. Delivering acute care from these units to people from different catchments and adjacent local government areas are peer workers and traditional staff from different disciplines, including but not limited to nurses and nurse educators. These units vary in local care models, restrictive practice rates, peer work involvement, and restrictive practice review approaches.

The control sites will be three declared adult inpatient units within two public hospitals in one LHD. Their total bed capacity is 84, and they are without high observation areas. Clinical input supported identifying these sites, considering the bed capacity and availability of high observation beds. One control site has no seclusion room, making it impossible to observe seclusion events or measure their duration. However, as detailed in the Analysis Plan section, the number of seclusion rooms and high observation beds will be considered for covariate balancing. Similar to the intervention sites, the three control units operate with varying levels of mental health practices and are multidisciplinary.

### Sample

Outcome change measurement and qualitative research within the evaluation depend on two forms of samples: daily aggregates of observed events and individual and nurse participants. Daily aggregates of restrictive practice and physical injury events will be used to analyse these outcomes. In contrast to a ‘per incident’ level, an aggregated approach helps prevent the false impression of completeness [20]. It enables a more accurate evaluation of potential reporting biases in real-world data. On the other hand, people receiving care and nurse participants will be involved in the qualitative strand and survey-based measures.

### Sample size

The sample size for this evaluation was determined using a combination of power analyses, prior research findings, and feasibility considerations. Both quantitative and qualitative approaches were employed.

For event-based analyses, Monte Carlo simulations using the simr package [59] and RStudio [60], were used to estimate the necessary duration of data collection. A Poisson mixed-effects model was applied to synthetic data replicating the initial implementation outcome parameters. To detect a 12% reduction in seclusion rates with 99.30% power, at least 3.60 months of data collection is required. To identify significant changes in physical restraint incidents, a 12-month period is necessary to achieve 90.60% power. In comparison, 10.8 months are required to detect differences in Code Black/ Assist events with 85.20% power. These calculations include a 20% buffer to account for potential attrition and were conducted with an alpha level of 0.50, aligning effect size assumptions with findings from the NSW Safewards evaluation [61]. The NSW Safewards evaluation was chosen as a reference, due to its high methodological quality and relevance to the same geographic and service context as this evaluation, being the only study identified in an overview of systematic reviews with such characteristics [5].

For survey-based measures, sample size estimations were conducted using G*Power 3 [62]. Based on power calculations, a minimum of 135 individual survey responses is required across intervention sites, incorporating a 30% margin for potential exclusions to achieve 95% power. Similarly, a minimum of 60 nurse surveys is necessary, maintaining the same power assumptions and buffer levels.

For the qualitative component, data will be collected through semi-structured interviews and focus groups. At least 15 individual interviews will be conducted at T2 and T4, with additional interviews, if necessary, until data saturation is reached. Additionally, three to five focus group discussions will be conducted per site, including six nurses involved in intervention implementation since baseline. These sample size decisions are informed by systematic reviews on qualitative research methodologies, indicating that such numbers are typically sufficient to achieve thematic saturation in studies using interviews [63] or focus groups [64].

### Recruitment and consent

The current evaluation will employ various recruitment strategies, with consent procedures tailored to each data collection activity. The implementation of Safe Steps follows an opt-in model, with recruitment proceeding through an asynchronous, multi-centre approach [65]. Each site independently decides when to implement Safe Steps, allowing for autonomy and flexibility, while ensuring consistency in the intended intervention across settings. Recruitment began at the first intervention site on 1st March 2024, with the latest site starting on 16th April 2024. Baseline data collection for the unit register, ims+, and eMeds data, covering the year before each site’s recruitment start date, commenced alongside recruitment. The final recruitment and data collection will conclude one year after the latest recruitment start, on 15th April 2025. At the time of the initial submission of the protocol for publication consideration, the study has reached its third time point.

For event-based analyses, the consent to access administrative data from unit registers and incident management systems was waived, because individual and nurse participant identifiers will not be gathered, nor will the data be re-identifiable. On the other hand, completion of the de-escalation log will serve as implied consent, and no identifying nurse details will be recorded. Identifying individual information will be gathered solely to help complete the logs, after which it will be removed and replaced with a randomly assigned study code before being shared with the first author.

For survey-based measures, peer worker-led surveys will be open to individuals over 18 who can consent to participate in the research. The only exclusion criteria are individuals receiving care with an identified need for an interpreter who refuse to use interpretation services adjacent to the unit. Increasing access to research participation with interpretation services was done with the consideration of guidance from the state-wide service that streamlines care pathways for culturally and linguistically diverse (CALD) individuals.

Each individual admitted to the intervention sites during data collection will receive a participant information sheet (PIS). Before discharge, people receiving care who meet the inclusion criteria will be approached by a peer worker and a nurse and provided with another copy of the PIS. To bring decision-making as close to the individual participant as possible, a nurse will assess an individual participant’s capacity to consent through the modified version of the University of California, San Diego Brief Assessment of Capacity to Consent [66]. Individual participants will be able to express interest in an interview through the final survey question, with identifying details quarantined from survey responses. Where needed, interpreters will be asked to support CALD participants during the capacity-to-consent assessment, survey completion, and interviews.

For the qualitative strand, nurses at intervention sites will be informed of the study during staff meetings and invited to participate in surveys and focus groups. They will receive the PIS and an email with a survey link. Focus group discussion recruitment will include information sessions, and nurses will be notified of focus group discussion timings via email. Written consent will be obtained before participation in focus group discussions. In contrast, nurses can indicate their consent through an item on the online survey.

### Measures

A battery of researcher-made and adapted standardised measures will be utilised in the evaluation. Adaptation was based on the context of acute mental health services and the research aims in which the evaluation occurs. Explicit permission was obtained for using and adapting the standardised measures, particularly those not openly available to the public. Best practice guidelines on psychometric validation of standardised measures will be followed [67,68].

#### Mental health nursing de-escalation logs.

The mental health nursing de-escalation log developed by a clinician (MF) in collaboration with another clinician (AT) has five sections: i) incident details, ii) aggression type, iii) interventions, iv) situational aggression levels, and v) outcomes, covering 51 variables. It records contextual information (date, location, number of staff involved), direction of aggression (e.g., individual to nurse), and lists interventions like the four Safe Steps and other non-relational, non-coercive de-escalation techniques. Situational aggression levels are rated on a 6-point scale from calm to physical aggression with weapons. Outcomes include restrictive practice use (e.g., seclusion, physical restraint, sedation via intramuscular injections), Code Black, time out, care level allocation, physical injuries, and incident management system reporting. Nurses will be trained to complete these logs, which they will contemporaneously cross-reference with unit data to minimise recall bias. Concurrent validation of the restrictive practice use outcomes will be attempted.

#### Fidelity checklist.

This checklist is an adaptation of the observational measure used in evaluating the implementation of the Safewards [20]. The adaptation concurs with the Safe Steps intervention and outcomes gathered through the de-escalation logs. Items 1–5 align with the implementation checks on the components of the Safe Steps, and items 6–10 are on the specific outcomes captured by the logs. There are no established norms that define full or partial implementation. However, the authors will consider higher scores on the first five items to indicate more vigorous implementation. In comparison, lower scores on the first five items suggest partial or minimal implementation.

#### Survey for People receiving care.

The survey is hosted on Qualtrics and will be completed on a touchscreen tablet. It begins with an introduction to the research and access to the PIS. Individual participants confirm their consent before completing 20 items of the adapted Consumer Evaluation of Mental Health Services Questionnaire [CEO-MHS Questionnaire; 69] and the 13-item Staff Action scale of the Modified Violence Prevention Climate [VPC-M-FR; 70]. Following the two psychometric measures, two researcher-made sentence-completion items towards individual’s feedback on something the acute mental health care unit has done well (plus)— “My experience would have been better if nurses...”—and something that the unit could use improvement (wish)— “The best things about nursing care in this service were…”—will be asked. Afterwards, direct questions will be used to gather demographic information. This information will not be used in the primary data analyses, although it will be summarised. Lastly, the survey ends with a page where individual participants may provide contact details should they wish to partake in a semi-structured interview. The administration of the people survey will follow the CEO-MHS questionnaire protocol to minimise response bias and emphasise Safe Steps’ value of autonomy. The scoring and interpretation of the standardised measures will follow their intended design and guidelines. Internal reliability estimation and factor structure validation of the standardised measures will be attempted.

#### Nurses survey.

The nurses in the intervention sites will be offered the opportunity to complete an online survey. The survey includes an introduction to the research and access to a PIS. After confirming their consent, participants will complete nine items of the Short Professional Quality of Life Scale [Short ProQOL; [71,72]; The Center for Victims of Torture, 2021; see www.ProQOL.org] and 70 items of the Genos Emotional Intelligence Inventory [73]. The study will collect demographic information through direct questioning. The demographic information will be reported as summaries, but not included in the primary data analysis. Finally, the survey concludes with a page where nurse participants can provide their e-mail addresses to gain an individualised emotionally intelligent workplace behaviour self-report. Only scores from the concise version (31 items) of the emotional intelligence inventory will be analysed for the Safe Steps evaluation. The scoring and interpretation of the standardised measures will follow their intended design and guidelines. Internal reliability estimation and factor structure validation of the standardised measures will be attempted.

#### Semi-structured interview schedule.

A researcher-made semi-structured interview schedule will be used with six main questions about admission, demographic information, ward experience, person-nurse relationships, restrictive practices experiences, and nurse actions.

#### Focus group discussion prompt.

A researcher-made focus group discussion prompt will be used with five main sections on individual aggression and emotional distress responses, individual relationships, needed de-escalation competencies, intervention implementation, and emotional intelligence.

### Procedures

The participating nurses will receive the training programme component of the Safe Steps. Nurse educators will support the opt-in-based implementation of the three components of the Safe Steps, ensuring that it is an integrated aspect of everyday practice in providing acute mental health care. Regular meetings, site visits, posters on the intervention’s key values, pull-up banners, coffee vouchers, and cheat sheet cards on the four steps will be implemented to support intervention uptake. Changes in nursing practices and measures of individual and nurse participants’ outcomes and experiences in the intervention sites will be collected over two years at various time points (see Fig 2). Restrictive practice and physical injury outcomes will be gathered prospectively from both intervention sites and control groups from administrative records, except for the de-escalation log. Outcomes from standardised measures and qualitative data will be collected prospectively only from people receiving care and nurse participants in the intervention sites. Data collection may cease due to unforeseen circumstances, such as reinstated COVID-19 restrictions.

### Analysis plan

Quantitative and qualitative analyses will be used to answer the evaluation’s objectives. Consultations with statisticians occurred for the range of statistical treatments employed in this evaluation. Various packages in R [74] and RStudio [60] will be maximised for the quantitative analyses, and Matplotlib [75] will be utilised for visualising outcomes. Each hypothesis will be supported if outcomes are statistically significant at p < .05.

Poisson mixed-effects models [56] will be used for count data and linear regression models for continuous outcomes, to determine whether these outcomes differ according to intervention implementation and time points. Data cleaning and validation, assumption checks, model diagnostics, and follow-up analyses will be undertaken. Negative binomial mixed models, zero-inflated models, and linear mixed-effect models may be used depending on the model’s diagnostics. Covariates in the mixed-effect models will include the acute care unit, calendar month, day, year, and the day within the study period for each daily event aggregate. Model selection will prioritise parsimony and explanatory strength. Inverse probability weighting (IPW) will be applied in the between- and within-group comparisons. Using the ipw package [76], IPW will be used to balance covariates, including but not limited to the average harm scores of daily aggregates, the number of high observation beds, the number of seclusion rooms, and other confounds (i.e., physical restraint and Code Blacks are closely interrelated, influencing each other’s occurrence or implementation [77]). Listed covariates for balancing will be used, subject to the data’s fit and the statistical model’s ability to achieve convergence in the IPW approach.

Hierarchical clustering analysis [78] will support the emergence of co-occurring mental health nursing response clusters, which will then be the fixed effect in modelling differential associations with restrictive practices, physical injuries, and situational aggression. This cluster and regression analysis constitutes the between-cluster comparison to identify the most active response clusters contributing most to Safe Steps’ overall impact.

Given the quality checks associated with national key performance indicators for mental health services, there is no expected missing data for the primary outcomes. However, log data may undergo multiple imputations under different missing data mechanisms [79] to minimise bias due to missing data. A day without log data will contribute to the missingness rate. Lastly, fidelity scores will be described statistically and plotted in a graph.

For survey-based measures, a one-way analysis of variance will be used to assess differences over time in people measures. In contrast, a paired t-test will examine changes in nurses’ measures over time. Missing data of not more than 20% will be imputed using multiple imputations [80]. Measures with less than 80% of the total items completed will be excluded. Non-parametric statistical models may be used if distributional assumptions are violated.

For the qualitative strand, pluses and wishes, transcribed interview responses, and focus group discussion data, will be analysed using NVivo 12 Pro software [81] and alternatively through a spreadsheet. An analytical process [82,83] that involves re-reading transcriptions for familiarisation, organising data, coding and condensed coding, and refining thematic results through a team approach, will be systematically led by the first author to integrate multiple perspectives. A codebook will be maintained [84]. Individual participants can review their responses for accuracy before analysis [85].

Machine learning techniques, including but not limited to random forest [86] and Boruta algorithm [87], will be maximised to undertake a feature analysis on the most important drivers of aggression and restrictive practice use. Qualitative log responses on triggers of de-escalation will be coded and considered in feature analyses.

### Ethics

In June 2022, an expert panel from one of the funding organisations provided the current evaluation with an initial scientific review (Ref: H22/47471). In March 2023, ethical approval and further scientific review were obtained from a committee in an LHD within NSW, Australia (2023/PID00297 - 2023/ETH00272). In May 2023, further ethical approval was received from the Southern Cross University Human Research Ethics Committee through the minimisation of duplication of ethical review application (Approval Number: 2023/069). In September and November 2024, amendments to the initial ethical approval and specific site approvals (ID 177103 & 187578) were completed.

### Research governance

The project steering committee (SC) will include representatives from each participating LHD, the State mental health branch, a clinical excellence body, Southern Cross University, and local Aboriginal Elders and health leads. Chaired by a senior mental health services manager, the SC will meet monthly, then bi-monthly, until completion, with key experts included as needed. The SC will report to the research governance committee, the executive directors of each LHD, and relevant patient safety and quality committees.

### Risk and adverse events

Potential discomfort from the surveys is minimal, but interviews may cause emotional distress when recalling inpatient care experiences. If necessary, support will be arranged as outlined in the PIS. Nurse participants may also experience inconvenience or discomfort from participation, and employee assistance will be arranged, if needed. Lastly, there is a possibility that individuals receiving care or nurse participants may disclose instances of poor care standards or inappropriate conduct that have implications for individual safety. If such disclosures occur, the researchers will report the details to the SC for attention and further action.

### Data governance

Third-party processes will manage contact details for individuals and nurse participants during recruitment and for generating nurses’ emotional intelligence reports, ensuring the research team cannot link identifying information, such as e-mail addresses, with participant data. Focus group and interview data will be de-identified by altering potentially identifiable details, such as sex, location, or events. Identifying demographic data from nurses and individual participants in the survey will not be shared in a form that could reveal individual identities, particularly in small samples. All administrative data and de-escalation logs will be de-identified and replaced with a random code before being shared with the first author, who will then aggregate the data to prevent further re-identification. The third-party process and data de-identification were intended to minimise bias in measuring outcomes. The data will be stored in a password-protected file on a secure, password-protected health service server for five years.

Due to the sensitive nature of the data, the two Human Research Ethics Committees that reviewed and approved this study have not granted ethical approval for its public sharing. However, if required, aggregate code books for qualitative analysis will be provided as supplementary material in peer-reviewed journal articles. Researchers seeking access to the data may direct inquiries to the Southern Cross University Human Research Ethics team at human.ethics@scu.edu.au.

### Publications

This study protocol was aimed at facilitating the assessment of bias in the selective reporting of results. The evaluation’s research findings will be submitted for publication in general and specialised peer-reviewed journals.

## Discussion

The Safe Steps implementation is timely and relevant. It brings structure and progression to therapeutic responding and deploys co-existing programme elements (i.e., training on model and restrictive practice reviews) in a phased and recurring approach over an extended time, addressing the gaps in many de-escalation training interventions [see 29, 88]. The Safe Steps incorporates cultural consultation, peer involvement, and lived experience advocacy in its design, implementation, and evaluation, which is distinctive in this space of mental health research [5]. Another strength of its content of care is tailoring practice through feedback, based on nurses’ successes in their therapeutic responses. Information on nurses’ practice of relational competence is gathered. It is then followed by sharing these successes during restrictive practice review meetings and reflections on them. This strengths-based feedback and monitoring is uncommon in many adult inpatient care reform initiatives [6]. In contrast, safe environments are supported when nurses are actively involved, and their voices and expertise are given primacy in these initiatives [89].

The Safe Steps evaluation is robust. It contributes to the limited knowledge base that discerns the most active co-occurring responses contributing to a programme or multi-component intervention’s overall impact. A significant limitation of complex interventions aimed at reducing restrictive practices is determining which elements contribute most to the effect, particularly as the impact of nurses’ therapeutic responses has not been evaluated in isolation. Rather, it is usual to see before-and-after evaluations of training interventions on these responses [see 29,88]. Moreover, control groups and statistical adjustments will be applied to address the absence of randomisation. Given that recruiting appropriate control groups and controlling for confounds is a well-documented challenge in acute care reform [26], this approach that balances covariates enhances this evaluation’s validity. This evaluation includes outcomes, such as sedation via intramuscular injection and physical injuries, that are rarely examined in de-escalation training interventions [29], helping to expand understanding of key indicators for evaluating mental health service performance in Australia [90]. The Safe Steps implementation and evaluation features a year-long period, allowing for the identification of both emergent and sustained impacts. This extended timeframe contrasts with most Safewards studies, which typically span only a few months [27].

The Safe Steps evaluation is open. The communication of this protocol addresses the gap in under-substantiated processes used to implement, replicate, and evaluate mental health nursing interventions. It also supports the emergence of mental health nursing practices, which are often invisible and may need more explicit role definitions [91]. More importantly, this study contributes to the scant evidence base on acute care efforts that openly communicate the use of capacity assessments that ensure participants are not forced to participate in research through informal coercion. Despite ethical approvals on safety-oriented studies, many evaluations do not explicitly mention in their recruitment and procedures any use of the capacity to consent in research assessments [see studies on ward atmosphere or climate in 27]. Finally, another strength of the Safe Steps’ evaluation is its use of both composite and disaggregated outcomes that will allow a comprehensive view of the intervention’s impact, while avoiding overrepresentation, masking effect bias, and loss of clinical interpretability of composite-only measures.

Identifying mechanisms of change in the Safe Steps is iterative. There is an effort to emerge the most active co-occurring responses that contribute to the Safe Steps’ overall impact, as well as the feedback and monitoring mechanism of the restrictive practice review meetings may help explain its effectiveness. However, the evaluation considers change mechanisms as key uncertainties [54] that can also be examined through the qualitative strand of research, including focus groups. As evidence builds, these uncertainties may change, requiring ongoing review at different stages of the research process. Nevertheless, understanding what works, for whom, how, and under what conditions is important for evaluating the Safe Steps in acute mental health units.

## Conclusions

The Safe Steps implementation and evaluation were intended to improve nurse-individual relationships and reduce restrictive practices in acute mental health units. The protocol behind these undertakings was aimed at promoting open and robust conduct of mental health research. The Safe Steps combines different components, like a user-friendly framework for de-escalation, value-based nurse training in therapeutic responding, and encouraging reflections on practice. Various approaches were considered to minimise research biases in the Safe Steps evaluation and implementation.
